# BEYOND FUNCTIONAL SUPPORT: PAID CAREGIVERS AND THE HEALTH OF OLDER ADULTS

**DOI:** 10.1093/geroni/igz038.789

**Published:** 2019-11-08

**Authors:** Jennifer M Reckrey, Barbara Bowers

**Affiliations:** 1 Icahn School of Medicine at Mount Sinai, New York, New York, United States; 2 University of Wisconsin-Madison, School of Nursing, Madison, Wisconsin, United States

## Abstract

This symposium will explore role that paid caregivers (e.g. home health aides, personal care attendants, and other direct care workers) play in the health and team-based healthcare of older adults living in the community. The large and growing workforce of paid caregivers witness the changes in health status, chronic health needs, and psychosocial stressors of the older adults they care for. Yet existing research on paid caregivers has largely been limited to workforce issues such as recruitment, retention, and job satisfaction. The unique potential of paid caregivers to impact the health of their clients remains largely unstudied. The first presenter will describe results from qualitative interviews with seriously ill older adults and their long-time paid caregivers that found that paid caregivers perform a wide variety of health-related tasks in the course of their routine care. The second presenter will describe results from focus groups with paid caregivers that found that though not a part of the official care plan, paid caregivers provided deliberate cognitive, emotional, and social care that sought to improve their clients’ “total” health. The third presenter will outline the rationale for and development of an educational intervention aiming to improve paid caregiver’s ability to provide care to patients with heart failure. Finally, the fourth presenter will discuss the limited role paid caregivers currently play in the healthcare team. She will then highlight key policy, educational, and clinical recommendations to promote further paid caregiver integration in the healthcare team.

